# Vaccination Drives Alveolar Macrophages Pool Remodeling via Training Resident Cells and Recruiting Monocyte‐Derived Cells to Defend Against Multidrug‐Resistant *Acinetobacter baumannii*

**DOI:** 10.1002/mco2.70887

**Published:** 2026-08-03

**Authors:** Xiaomin Zhang, Yangyang Zhou, Chuanying Xiang, Ning Wang, Yan Li, Kai Chen, Yu Xie, Hong Yang, Xiangcheng Sun, Yun Shi

**Affiliations:** ^1^ Institute of Biopharmaceuticals West China Hospital Sichuan University Chengdu Sichuan China

**Keywords:** *Acinetobacter baumannii* (*A. baumannii*), trained immunity, alveolar macrophages, multidrug‐resistant (MDR) bacteria, intranasal vaccination

## Abstract

Alveolar macrophages (AMs) serves as a frontline innate barrier against pulmonary bacterial invasion. The AM pool comprises tissue‐resident AMs (TR‐AMs) and monocyte‐derived AMs (Mo‐AMs); yet how vaccination remodels the mixed AM pool for long‐term antimicrobial defense against multidrug‐resistant bacteria remains poorly understood. In this work, we applied intranasal inactivated whole‐cell (IWC) vaccination against *Acinetobacter baumannii* and systematically dissected AM population dynamics at cellular and molecular levels. Vaccination reshaped the AM pool by recruiting CD11B^+^CD13^+^ Mo‐AMs that gradually acquire a TR‐AMs‐like phenotype and training TR‐AMs via sustained transcriptional adaptations. Functionally, the IWC‐remodeled AM pool exhibited enhanced antigen presentation, TNF‐α secretion, and phagocytosis, accompanied by metabolic rewiring, thereby conferring durable protection against *A. baumannii* infection. ATAC‐seq revealed immune‐related chromatin remodeling and enrichment of transcription factors, including ETS, IRF, and bZIP family members. Similar AM pool remodeling was observed following immunization with IWC vaccines against *Pseudomonas aeruginosa* and *Klebsiella pneumoniae*, suggesting parallel innate responses across multiple common respiratory bacterial vaccines. Collectively, our study delineates the single‐cell transcriptional and epigenetic landscapes of vaccine‐remodeled AM subsets, providing mechanistic insights to guide the development of AM‐targeted prophylactic vaccines against multidrug‐resistant *A. baumannii*.

## Graphical abstract

Under homeostatic conditions, the lungs primarily harbor tissue‐resident alveolar macrophages (TR‐AMs). Intranasal immunization with *A. baumannii* IWC remodels the alveolar macrophage pool via two complementary mechanisms: recruitment and progressive differentiation of CD11B^+^CD13^+^ monocyte‐derived AMs, together with direct functional and epigenetic reprogramming of resident TR‐AMs. This dual remodeling imprints long‐term trained immunity in the lung, enhancing MHC II expression, cytokine secretion, and bacterial phagocytosis to confer durable protection against subsequent MDR‐AB challenge.

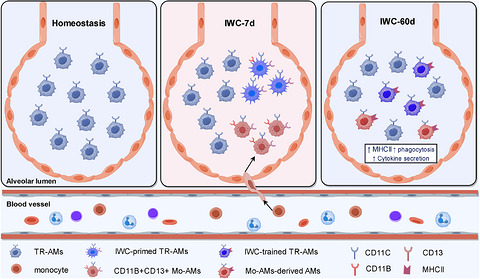

## 1 Introduction

The alveolar macrophage (AM) pool is a heterogeneous population of immune cells that populate the alveolar interface, serving as pivotal sentinels of lung immunity. Positioned at this critical barrier, they orchestrate phagocytosis, inflammatory modulation, and tissue homeostasis, adapting dynamically to environmental cues and pathogenic challenges [1].

The AM pool comprises two key subsets with distinct ontogenies, molecular features, and transcriptional regulatory networks: tissue‐resident AMs (TR‐AMs) and monocyte‐derived AMs (Mo‐AMs) [2, 3, 4]. Under steady‐state conditions, TR‐AMs originate from embryonic yolk sac‐derived progenitors, establishing a long‐lived population maintained by self‐renewal in adulthood [2]. They are defined by signature markers CD11C, SIGLECF, MARCO, and MRC1, with a transcriptomic profile enriched in homeostatic repair and immune tolerance. Their development is governed by GM‐CSF, which activates the core transcription factor (TF) PU.1 [5]. In contrast, Mo‐AMs derive from circulating monocytes, characterized by CD11B expression, and their differentiation depends on sustained interactions with the tissue microenvironment. Following lung injury or infection, TR‐AMs may remain unchanged, acquire new functionality, or be replaced by Mo‐AMs, leading to a dynamic reshaping of the AM pool to meet the demands of the immune response [6].

Beyond their classical roles, AMs exhibit “trained immunity”: a form of innate memory driven by metabolic and epigenetic reprogramming, enabling enhanced responses to subsequent challenges [7, 8]. Unlike adaptive immunity, this process involves broad transcriptional shifts, heightened cytokine secretion, and metabolic rewiring, as observed in AMs after infections with influenza, SARS‐CoV‐2, or *Streptococcus pneumoniae* [3, 9, 10, 11]. Notably, TR‐AMs can undergo intrinsic reprogramming independently of Mo‐AMs [9, 10, 12, 13], while recruited Mo‐AMs may develop distinct phenotypes [3, 14], highlighting the AM pool's dynamic potential for context‐specific adaptation.

Given this ability of infections to induce trained immunity in AMs, vaccination may exploit these similar mechanisms to enhance long‐term immune protection against respiratory pathogens. Studies indicate that vaccination, such as BCG, live attenuated *Pseudomonas aeruginosa* vaccines, and influenza‐vectored COVID‐19 vaccines, can induce trained immunity in AMs [15, 16, 17]. However, how vaccination remodels the AM pool and the distinct roles of TR‐AMs versus Mo‐AMs in vaccine‐induced immunity remain controversial. TR‐AMs have been shown to acquire a memory phenotype independently of Mo‐AMs, as observed in BCG and *P. aeruginosa* vaccines [15, 16], which induce upregulated expression of MHC II, CD86, and iNOS [15, 18]. By contrast, influenza‐vectored COVID‐19 vaccines predominantly drive Mo‐AM recruitment, suggesting vaccine‐specific differences in AM pool remodeling [17].

*Acinetobacter baumannii* (*A. baumannii*), a multidrug‐resistant (MDR) Gram‐negative bacteria, poses a great threat in hospital settings, causing lethal pneumonia with limited therapeutic options [19]. Our prior work showed that intranasal immunization with inactivated whole‐cell (IWC) *A. baumannii* confers rapid protection against MDR *A. baumannii* (MDR‐AB) via AM‐trained immunity [20], but the underlying pool dynamics—whether protection relies on TR‐AMs training, Mo‐AMs recruitment, or both—remain unclear. Addressing this knowledge gap is essential for optimizing vaccine design and harnessing AM‐trained immunity for respiratory infection control.

Here, we used single‐cell RNA sequencing (scRNA‐seq) and ATAC‐seq to dissect how IWC vaccination remodels the AM pool. We demonstrated that vaccination drives coordinated changes: recruiting a CD11B^+^CD13^+^ Mo‐AM subset that undergoes TR‐AM‐like conversion, while directly training TR‐AMs via sustained transcriptional and epigenetic adaptations. These findings provide mechanistic insights into vaccine‐induced AM pool remodeling in the context of *A. baumannii* vaccination, offering a cellular and molecular framework to design AM‐targeted vaccine against MDR‐AB.

## 2 Results

### 2.1 Vaccine‐Induced Innate Immunity Confers Long‐Lasting Protection Against MDR‐AB Pneumonia

**FIGURE 1:**
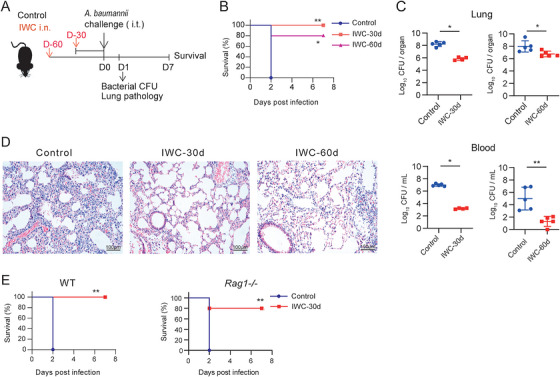
Intranasal IWC vaccination elicits long‐lasting protection against MDR‐AB pneumonia via innate immunity. (A) Schematic overview of the experimental protocol. Mice received a single intranasal immunization with IWC vaccine, followed by intratracheal challenge with a lethal dose of *A. baumannii* at 30 or 60 days post‐immunization. (B) Kaplan–Meier survival curves for control mice and mice challenged 30 days (IWC‐30d) or 60 days (IWC‐60d) post‐immunization (*n* = 5). (C) Bacterial loads in the lungs and blood were measured at 24 h post‐infection in control and IWC‐immunized mice challenged at 30 days and 60 days. (D) Representative hematoxylin and eosin (H&E)‐stained lung sections from control, IWC‐30d, and IWC‐60d groups at 24 h post‐infection. Scale bar, 100 µm. (E) Survival curves of wild‐type and *Rag1^−^* ^/^ *^−^* mice immunized with IWC for 30 days and challenged with *A. baumannii* (*n* = 5). Survival data were analyzed using the log‐rank test, and differences in bacterial loads were assessed by the Mann–Whitney *U* test. ^*^ *p* < 0.05, ^**^ *p* < 0.01, compared with control group. Data are representative of two independent experiments.

We first validated the durability of protection conferred by our intranasal IWC vaccine, which we previously showed elicits rapid protection via AM‐trained immunity [20]. C57BL/6 mice were immunized with a single intranasal IWC immunization and were challenged intratracheally with a lethal dose of MDR‐AB at 30 or 60 days post‐immunization (Figure 1A). Strikingly, 100% of immunized mice survived the challenge at 30 days, and 80% survived at 60 days—compared to 100% death in control mice (Figure 1B). This protection was accompanied by markedly decreased bacterial loads in the lungs and blood of vaccinated mice (Figure 1C) and as well as alleviated lung tissue damage and inflammatory infiltration at 24 h post‐infection (Figure 1D). Critically, *Rag1* ^−/−^ mice (deficient in adaptive immunity) exhibited comparable long‐term survival (Figure 1E), confirming that innate immunity—rather than B/T cell responses—mediates this durable protection.

### 2.2 IWC Immunization Drives Dynamic Remodeling of the AM Pool

**FIGURE 2:**
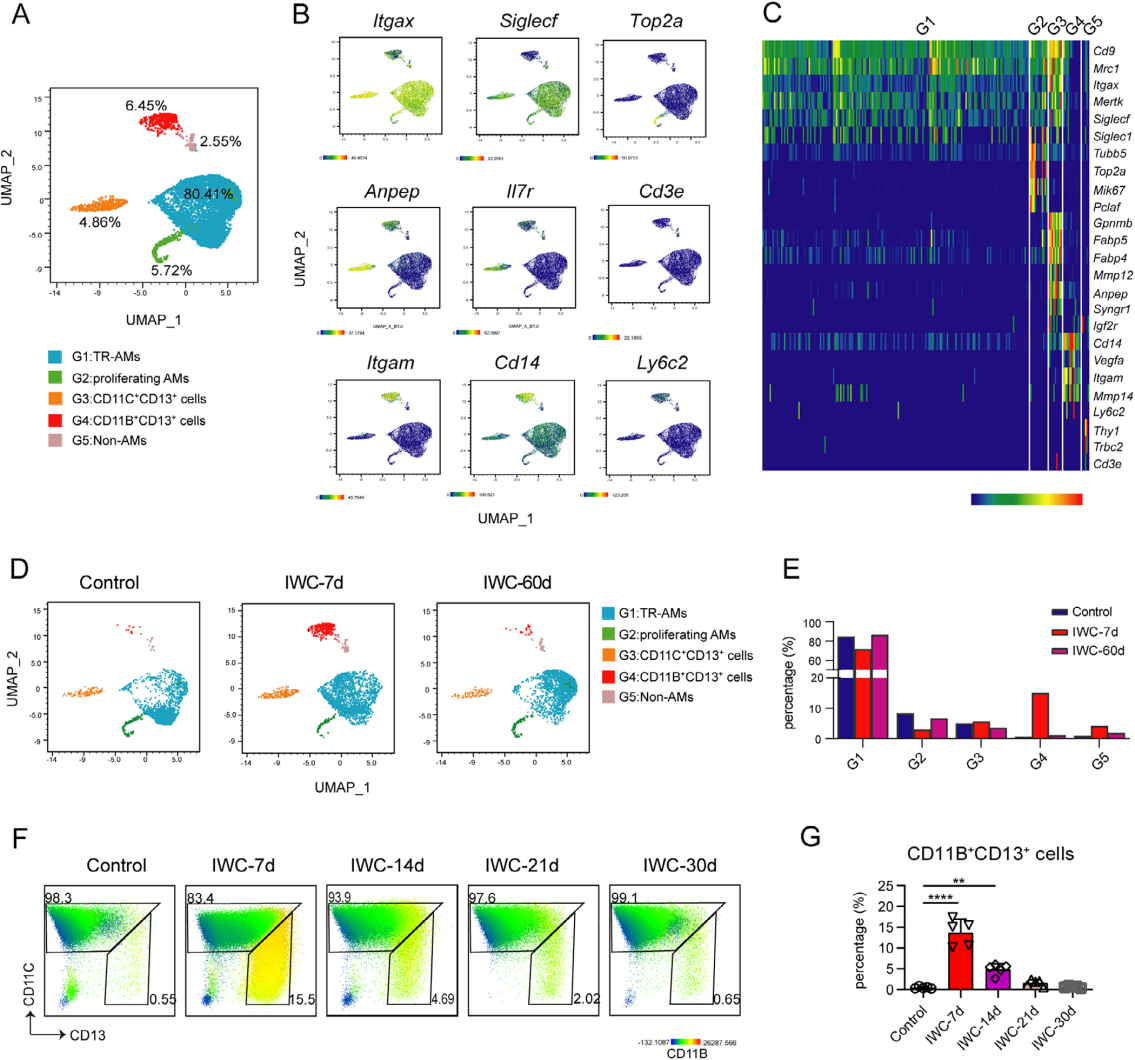
Changes in the AM pool composition following intranasal IWC immunization. (A) UMAP projection of 6745 single‐cell transcriptomes obtained from BALF of control and IWC‐immunized mice at 7 and 60 days post‐immunization, identifying five cell Groups (G1–G5). Group 1 (G1): classical TR‐AMs, characterized by Itgax^+^, Siglecf^+^. Group 2 (G2): proliferating AMs, marked by Top2a^+^, Mki67^+^. Group 3 (G3): CD11C^+^CD13^+^ cells expressing Itgax^+^, Anpep^+^ (encoding CD13). Group 4 (G4): CD11B^+^CD13^+^ cells, marked by *Itgam* ^+^ *Anpep* ^+^. Group 5 (G5): non‐AM immune cells. (B) UMAP plots displaying the expression patterns of key marker genes for each cell group. (C) Heatmap depicting signature gene expression across the five cell groups. (D) UMAP representations illustrating the distribution of cell groups in BALF samples from control, IWC‐7d, and IWC‐60d mice. (E) Bar graph indicating the relative proportions of each cell group in the control and IWC‐immunized groups. (F) Representative flow cytometry plots showing CD11C and CD13 expression in BALF cells from control and IWC‐immunized mice at Days 7, 14, 21, and 30 post‐immunization. Dot color reflects CD11B expression intensity, with the color scale shown in the lower right. All events are pre‐gated on non‐monocytes shown in Figure S1D. (G) Quantification of the percentage of CD11B^+^CD13^+^ cells over the time course (*n* = 5 per group). Data are presented as mean ± SD. Statistical significance was determined by one‐way ANOVA with Tukey's multiple comparisons test; ^**^ *p* < 0.01, ^****^ *p* < 0.0001.

To define how the AM pool contributes to vaccine protection, we performed scRNA‐seq on AMs isolated from bronchoalveolar lavage fluid (BALF) of control mice and IWC‐immunized mice at 7 and 60 days post‐immunization (key time points for early and long‐term protection). After quality control (QC; Figure S1A), 6745 cells were analyzed (1862 control; 2624 IWC‐7d; 2259 IWC‐60d). Unbiased Uniform Manifold Approximation and Projection (UMAP) clustering identified five transcriptionally distinct cell populations, which we annotated based on canonical marker genes of AMs and published scRNA‐seq datasets (Figure 2A and Figure S1B). Specifically, Group 1 (G1) was defined as classical TR‐AMs, characterized by high expression of AM lineage‐defining markers including Itgax (encoding CD11C), Siglecf, Mrc1 (encoding CD206), and Siglec1 (CD169). Group 2 (G2) was proliferating AMs, marked by the expression of cell‐cycle‐related genes including *Top2a, Mki67*, and *Pcla*. Group 3 (G3) was defined as CD11C^+^CD13^+^ cells, expressed Itgax, Anpep (encoding CD13), Il7r, Gpnmb, Fabp4, Fabp5, Syngr1, and Mmp12. Group 4 (G4) was defined as CD11B^+^CD13^+^ cells, exhibiting high levels of Itgam (encoding CD11B), Anpep, Ly6c2, Cd14, Mmp14, and Vegfa, alongside low Itgax expression—hallmarks of monocyte‐derived macrophages. Group 5 (G5) was non‐AM immune cells, predominantly T cells, as confirmed by specific expression of T cell markers Cd3e, Trbc2, and Thy1 (Figure 2B,C and Figure S1C).

Quantitative analysis of cell proportions across groups revealed a significant but transient increase in CD11B^+^CD13^+^ cells at 7 days post‐immunization, with proportions returning to baseline by 60 days (Figure 2D,E).

Among the differentially expressed genes (DEGs) in G4 identified by scRNA‐seq, CD13 was selected as a candidate marker for flow cytometry validation due to its cell surface expression and robust upregulation in this subset. Flow cytometry analysis of BALF cells showed a marked increase in CD11C^−^CD13^+^ cells at Day 7 post‐IWC immunization. Critically, color‐coded CD11B expression intensity on the dot plots revealed that these CD11C^−^CD13^+^ cells co‐expressed high levels of CD11B, consistent with the transcriptomic profile of the increased CD11B^+^CD13^+^ cells population (Figure 2F). The flow cytometry gating strategy is shown in Figure S1D. Time‐course quantification further demonstrated that the frequency of CD11B^+^CD13^+^ cells peaked at Day 7, then gradually declined by Days 14, 21, and 30, returning to baseline levels by Day 30 (Figure 2F,G). These findings demonstrate that IWC immunization triggers dynamic remodeling of the AM pool by recruiting CD11B^+^CD13^+^ cells, which gradually decline over time.

### 2.3 CD11B^+^CD13^+^ Cells Represent Transitional Mo‐AMs

**FIGURE 3:**
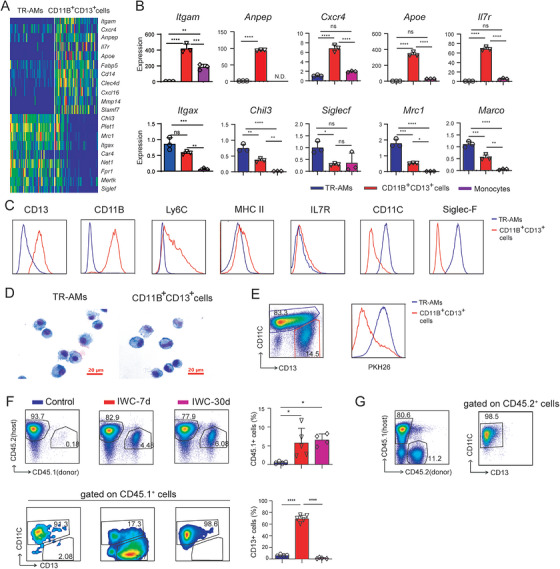
CD11B^+^CD13^+^ cells represent transitional Mo‐AMs. (A) Heatmap showing top differentially expressed genes between TR‐AMs and CD11B^+^CD13^+^ cells identified by scRNA‐seq. (B) qRT‐PCR validation of signature gene expression in FACS‐sorted TR‐AMs, CD11B^+^CD13^+^ cells, and circulating monocytes at Day 7 post‐IWC immunization (*n* = 3). Data expressed as mean ± SD. ^*^, *p*<0.05, ^**^, *p* < 0.01, ^***^, *p* < 0.001, ^****^, *p* < 0.0001, ns, not significant, analyzed by one‐way ANOVA. N.D., not detected. (C) Flow cytometry profiling of marker expression in CD11B^+^CD13^+^ cells compared to TR‐AMs from BALF at Day 7 post‐immunization. (D) Giemsa staining of FACS‐sorted CD11B^+^CD13^+^ cells and TR‐AMs isolated from BALF. (E) TR‐AMs labeled intratracheally with fluorescent phagocytic cell dye PKH26‐PCL prior to IWC immunization. Representative flow cytometry plots showing PKH26 signal in TR‐AMs and CD11B^+^CD13^+^ cells at Day 7 post‐immunization. (F) Lung‐shielded bone marrow chimera experiment using CD45.2^+^ recipient mice transplanted with CD45.1^+^ bone marrow cells. Six weeks post‐transplantation, mice were immunized intranasally with IWC. BALF was collected at Days 7 and 30 post‐immunization and analyzed by flow cytometry to assess the presence and phenotype of donor‐derived (CD45.1^+^) cells (*n* = 4–5). (G) CD11B^+^CD13^+^ cells were FACS‐sorted from BALF of IWC‐immunized CD45.2^+^ donor mice at Day 5 and transferred intratracheally into congenic CD45.1^+^ recipient mice. BALF was collected at 25 days post‐transfer and analyzed via flow cytometry to assess the presence and phenotype of grafted CD45.2^+^ cells.

scRNA‐seq profiling identified a CD11B^+^CD13^+^ cell cluster that was transcriptionally distinct from classical CD11B^−^CD11C^+^ TR‐AMs, as visualized by differential marker expression (Figure 3A). To verify these transcriptomic differences and characterize the identity of this population, we performed quantitative real‐time PCR (qRT‐PCR) on sorted CD11B^−^CD11C^+^ TR‐AMs, CD11B^+^CD13^+^ cells from the BALF, and circulating monocytes as a developmental reference control at Day 7 post‐intranasal IWC immunization. Consistent with scRNA‐seq data, CD11B^+^CD13^+^ cells exhibited a unique transcriptional signature distinct from both TR‐AMs and circulating monocytes (Figure 3B and Figure S2A). Compared with TR‐AMs, CD11B^+^CD13^+^ cells expressed significantly higher levels of monocyte and monocyte‐derived macrophage markers, including Itgam, Ly6c, Ccr2, Cxcr4, and Csf1r, as well as distinctive signature genes such as *Anpep*, *Apoe*, and *Il7r*. In contrast, these cells showed lower expression of canonical TR‐AM markers, such as Itgax, Chil3, Siglecf, Mrc1, and Marco (Figure 3B and Figure S2A).

Flow cytometry further confirmed that Day 7 CD11B^+^CD13^+^ cells in BALF displayed high levels of CD11B, CD13, Ly6C, MHC II, and IL7R, with lower CD11C and Siglec‐F compared to TR‐AMs (Figure 3C). Notably, CD13 (Anpep) was undetectable in circulating monocytes (N.D.) by qRT‐PCR and flow cytometry (Figure 3B and Figure S2B), suggesting it serves as a unique marker for CD11B^+^CD13^+^ cells. Giemsa staining revealed classical AM morphology in TR‐AMs, while CD11B^+^CD13^+^ cells exhibited macrophage‐like features (Figure 3D). To determine tissue localization of these cells, we performed in vivo antibody labeling: mice received intravenous PE‐Cy7‐conjugated anti‐CD45 to label circulating cells and intratracheal BV605‐conjugated anti‐CD45 to label airway cells. Flow cytometric analysis of lung suspensions showed that CD11B^+^CD13^+^ cells were predominantly BV605^+^, confirming their alveolar localization similar to TR‐AMs (Figure S2C).

To trace the origin of CD11B^+^CD13^+^ cells, TR‐AMs were selectively labeled via intratracheal administration of PKH26‐PCL dye before IWC immunization. At Day 7 post‐immunization, TR‐AMs remained PKH26‐positive, whereas CD11B^+^CD13^+^ cells were predominantly PKH26‐negative (Figure 3E), indicating that CD11B^+^CD13^+^ cells were not derived from pre‐existing TR‐AMs. Collectively, these results establish that CD11B^+^CD13^+^ cells represent Mo‐AMs with a transitional differentiation state.

Mo‐AMs might either undergo apoptosis and disappear following inflammation resolution or adapt to the alveolar niche for long‐term persistence [3, 21]. To track the long‐term fate of CD11B^+^CD13^+^ cells post‐IWC immunization, we generated lung‐shielded bone marrow chimeras by transplanting CD45.1^+^ bone marrow cells into irradiated CD45.2^+^ recipients. Six weeks post‐transplantation, mice were immunized intranasally with IWC, and BALF cells were analyzed by flow cytometry. CD45.1^+^ cells were detectable at both Day 7 and Day 30 post‐immunization, indicating recruited Mo‐AMs were long‐lasting in the alveolar niche (Figure 3F). At Day 7, CD45.1^+^ cells primarily exhibited a CD11B^+^CD13^+^CD11C^−^ phenotype, transitioning to CD11B^−^CD13^−^CD11C^+^ by Day 30 (Figure 3F). Adoptive transfer of sorted CD11B^+^CD13^+^ cells from IWC‐immunized CD45.2^+^ donors into congenic CD45.1^+^ recipients further confirmed that these cells persisted for up to 30 days and underwent a phenotypic conversion toward a TR‐AM‐like profile, characterized by downregulation of CD11B and CD13, and upregulation of the canonical TR‐AM marker CD11C (Figure 3G). Collectively, these findings demonstrate that CD11B^+^CD13^+^ cells, recruited from circulating monocytes following IWC immunization, represent transitional Mo‐AMs that have entered the alveolar space but retain monocyte‐like features and have not fully matured into canonical TR‐AMs; they gradually acquire a TR‐AM‐like phenotype and serve as precursor cells that contribute to the long‐term AM pool in response to pulmonary vaccination.

### 2.4 Modulation of TR‐AMs by IWC Immunization

**FIGURE 4:**
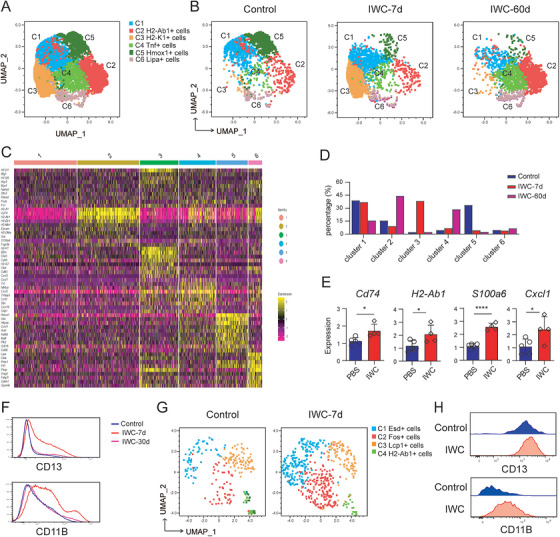
Modulation of TR‐AMs by IWC immunization. (A) UMAP visualization of six transcriptionally distinct subclusters identified by re‐clustering of TR‐AMs (Group 1) from scRNA‐seq data in Figure 2A. (B) UMAP plots showing the distribution of TR‐AMs clusters in BALF samples from control, IWC‐7d, and IWC‐60d groups. (C) Heatmap illustrating the top DEGs for each TR‐AM subcluster. (D) Quantification of the relative proportion of each TR‐AM subcluster in control and IWC‐immunized groups at Day 7 and Day 60 post‐immunization. (E) qRT‐PCR analysis of gene expression in TR‐AMs isolated at Day 30 post‐IWC immunization. Data are presented as mean ± SD. *n* = 4–5 mice per group. ^*^, *p* < 0.05, ^****^, *p* < 0.0001, unpaired two‐tailed Student's *t*‐test. (F) Flow cytometry histograms showing CD13 and CD11B expression on PKH26‐labeled TR‐AMs at Day 7 and Day 30 post‐IWC immunization. (G) UMAP plots depicting four clusters of TR‐AMs following in vitro stimulation with IWC for 7 days. (H) Flow cytometry histograms showing CD13 and CD11B expression in control and IWC‐treated TR‐AMs after 7 days of in vitro culture.

scRNA‐seq analysis of BALF cells showed that TR‐AMs (G1) underwent dynamic phenotypic modulation following IWC immunization (Figure 2D). To better characterize these changes, we performed re‐clustering of TR‐AMs, identifying six transcriptionally distinct subclusters (Figure 4A). Notably, the proportional distribution of these subclusters differed dramatically among control, IWC‐7d, and IWC‐60d groups (Figure 4B,D). In control mice, TR‐AMs primarily clustered into Cluster 1 and Cluster 5 (Figure 4B,D). Cluster 5 was characterized by genes associated with metabolic regulation, including *Hmox1, Ubo, Hilpda, Ccnl1, Kat6b*, and *Cd36* (Figure 4C and Table S1). At Day 7 post‐IWC immunization, Cluster 3 was significantly increased, while Cluster 5 was markedly reduced. (Figure 4B,D). Cluster 3 exhibited high expression of immune activation‐related genes, including *H2‐K1*, *B2m*, *Chil3*, *Cybb*, *H2‐Q7*, and *Cxcl1* (Figure 4C). By Day 60, the TR‐AM population further transitioned to Cluster 2 and Cluster 4 (Figure 4B,D). Cluster 2 exhibited high expression of antigen‐processing and presentation genes (*H2‐Ab1*, *Cd74*, *H2‐Eb1*, *Epcam*, and *Fcgr2b*), while Cluster 4 expressed inflammatory mediators *(Tnf*, *Cxcl1*, *Nfkb1a*, *Cxcl2*, and *Tnfaip3*) (Figure 4C). Cluster 6, a minor cluster, remained unchanged post‐immunization (Figure 4D). To validate these transcriptomic changes, TR‐AMs were isolated at Day 30 post‐immunization and subjected to qRT‐PCR analysis. We observed increased expression of *CD74*, *H2‐Ab1*, *S100a6*, and *Cxcl1* (Figure 4E), confirming the phenotypic shift observed in Cluster 2 and Cluster 4 from scRNA‐seq data (Figure 4B,C). Given the above finding that CD11B^+^CD13^+^ cells convert into CD11B^−^CD11C^+^ AMs over time, we hypothesized that this transition might contribute to the observed upregulation of activation‐related genes in TR‐AMs. To directly assess the effect of IWC on resident TR‐AMs, we labeled TR‐AMs with PKH26 dye before immunization. At Day 7 post‐immunization, PKH26^+^ TR‐AMs exhibited elevated CD11B and CD13 expression, which returned to baseline by Day 30, indicating a direct modulation of the TR‐AM phenotype by IWC (Figure 4F). To further confirm that IWC directly induces phenotypic changes in TR‐AMs independent of other immune interactions, we isolated TR‐AMs from the BALF of naïve mice and stimulated them in vitro with IWC for 7 days, followed by scRNA‐seq analysis. Clustering analysis identified four distinct clusters, showing that IWC treatment reshaped TR‐AMs’ transcriptomic profile (Figure 4G and Table S2). Furthermore, flow cytometry confirmed that IWC‐treated TR‐AMs expressed significantly higher levels of CD11B and CD13 compared to untreated controls (Figure 4H). Collectively, these findings demonstrate that IWC immunization directly modulates TR‐AMs.

### 2.5 IWC Immunization Enhances the Functional and Metabolic State of the AM Pool

**FIGURE 5:**
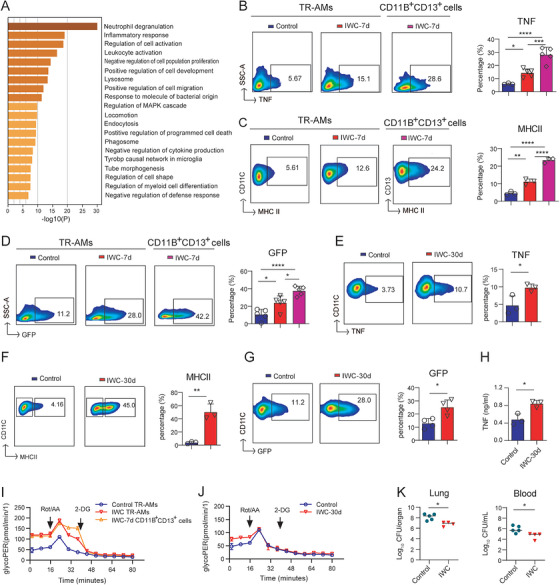
IWC immunization enhances the functional and metabolic state of the AM pool. (A) Gene set enrichment analysis of signature genes from CD11B^+^CD13^+^ cells isolated from IWC‐immunized mice at Day 7 post‐immunization. Enriched terms are ranked by −log_10_ (*p* value). (B and C) Representative flow cytometry plots and quantification of intracellular TNF‐producing cells (B) and MHC II‐expressing cells (C) in control TR‐AMs, IWC‐trained TR‐AMs, and CD11B^+^CD13^+^ Mo‐AMs sorted from BALF at Day 7 post‐IWC immunization. Data are shown as mean ± SD; ^*^, *p* < 0.05, ^**^, *p* <0.01, ^***^, *p* < 0.001, ^****^, *p* < 0.0001, determined by ordinary one‐way ANOVA. (D) Representative flow cytometry plots and quantification of *A. baumannii*‐GFP^+^ cells in control TR‐AMs, IWC‐trained TR‐AMs, and CD11B^+^CD13^+^ Mo‐AMs from IWC‐immunized mice at Day 7 post‐immunization (*n* = 5 mice per group). (E–G) Representative flow cytometry plots and quantification of intracellular TNF production (E), MHC II expression (F), and phagocytic uptake of GFP^+^ *A. baumannii* (G) in control and IWC‐trained AMs at Day 30 post‐IWC immunization (*n* = 3–4 mice per group). Data are shown as mean ± SD; ^*^, *p* < 0.05, ^**^, *p* < 0.01, analyzed by Student's *t*‐test. (H) AMs isolated from control or IWC‐immunized mice at Day 30 were restimulated ex vivo with IWC for 2 h. TNF production in culture supernatants was measured by ELISA (*n* = 3 per group). ^*^, *p* < 0.05, Student's *t*‐test. (I and J) Glycolytic activity of sorted AMs assessed by Seahorse XF Glycolytic Rate Assay. (I) Glycolytic proton efflux rate (glycoPER) over time in control TR‐AMs, IWC‐trained TR‐AMs, and CD11B^+^CD13^+^ Mo‐AMs at Day 7 post‐IWC immunization. (J) glycoPER profiles in control and IWC‐trained TR‐AMs at Day 30. Rotenone/antimycin A (Rot/AA) and 2‐deoxy‐D‐glucose (2‐DG) were injected at the indicated time points. (K) Bacterial burdens in the lung and blood of recipient mice following adoptive transfer of sorted AMs. AMs (CD11C^+^CD11B^−^) isolated from control or Day 30 IWC‐immunized donors were adoptively transferred to naïve recipients, followed by *A. baumannii* challenge 4 h later. Bacterial loads were quantified at 14 h post‐infection (*n* = 4–5 mice per group). ^*^, *p* < 0.05, Mann–Whitney *U* test.

Having established that IWC immunization alters the composition and phenotype of the AM pool, we next examined whether these changes influence their functional properties. scRNA‐seq analysis revealed that CD11B^+^CD13^+^ Mo‐AMs were enriched in pathways related to inflammatory responses, lysosomal activity, endocytosis, and phagocytosis (Figure 5A). Similarly, TR‐AMs exhibited distinct functional shifts following IWC immunization: at Day 7, they clustered into Cluster 3, showing enrichment in inflammatory and phagocytic pathways (Figure S3A). By Day 60, TR‐AMs predominantly resided in Cluster 2 and Cluster 4; Cluster 2 was enriched in antigen processing and presentation via MHC class II, while Cluster 4 displayed enrichment in cytokine response genes (Figure S3B,C). These findings suggested functional reprogramming of the AM pool, enhancing both immune responsiveness and bacterial clearance capacity following IWC immunization. Flow cytometry of TR‐AMs post‐immunization revealed that, at Day 7, CD11B^+^CD13^+^ Mo‐AMs exhibited a higher frequency of TNF‐producing and MHC II‐expressing cells compared to both control and IWC‐trained TR‐AMs (Figure 5B,C). In addition, IWC‐trained TR‐AMs also showed increased TNF and MHC II expression relative to control TR‐AMs, indicating functional activation in both AM subsets (Figure 5B,C).

Since enhanced phagocytosis is a hallmark of trained immunity and a key function for bacterial elimination, we evaluated bacterial uptake following IWC immunization. At Day 7, flow cytometry of BALF demonstrated that CD11B^+^CD13^+^ Mo‐AMs had a significantly higher proportion of GFP^+^ cells compared to TR‐AMs, suggesting a stronger ability to engulf *A. baumannii* (Figure 5D). Notably, GFP^+^ TR‐AMs were also increased in IWC‐immunized mice compared to controls, confirming enhanced phagocytic capacity in both populations (Figure 5D). In vitro modeling of trained TR‐AMs confirmed these findings, as IWC‐trained TR‐AMs exhibited significantly greater uptake of GFP‐labeled *A. baumannii* compared to untreated controls (Figure S3D). Importantly, these functional adaptations were sustained up to 30 days post‐immunization. IWC‐trained AMs maintained elevated TNF secretion (Figure 5E), sustained MHC II expression (Figure 5F), and enhanced phagocytic capacity relative to controls (Figure 5G). Moreover, ex vivo restimulation of AMs isolated 30 days post‐immunization showed increased TNF secretion (Figure 5H), supporting the persistence of a trained immunity phenotype with heightened responsiveness to secondary stimulation.

Notably, enhanced glycolysis is a well‐characterized hallmark of trained immunity, serving as the key metabolic basis to support the enhanced functional responses of trained immune cells [22]. To explore the metabolic reprogramming of AMs following IWC immunization, we assessed metabolic status in sorted AMs using Seahorse extracellular flux analysis. At Day 7 post‐immunization, both IWC‐trained TR‐AMs and CD11B^+^CD13^+^ Mo‐AMs exhibited significantly elevated basal glycolysis and compensatory glycolysis compared to control TR‐AMs. IWC‐trained TR‐AMs displayed the highest glycolytic activity, particularly in compensatory glycolysis, mirroring their robust functional activation (Figure 5I and Figure S3E). At Day 30 post‐IWC immunization, IWC‐trained AMs showed significantly elevated basal glycolysis compared to control TR‐AMs (Figure 5J and Figure S3F), where compensatory glycolysis was comparable between the two groups. These metabolic changes align with their trained immune phenotype, including enhanced inflammatory, antigen‐presenting, and phagocytic functions.

Further, to determine whether IWC‐trained AMs contribute to protection against infection, we performed adoptive transfer experiments. Sorted CD11C^+^CD11B^−^ TR‐AMs from mice 30 days post‐IWC immunization were transferred into naïve recipients, followed by challenge with *A. baumannii*. Recipients of IWC‐trained AMs showed significantly reduced bacterial burdens in the lungs and bloodstream, demonstrating their protective capacity (Figure 5K). Collectively, these findings indicate that IWC immunization functionally reprograms the AM pool with enhanced antimicrobial and immune‐modulatory functions.

### 2.6 IWC Immunization Induces Epigenetic Reprogramming in the AM Pool

**FIGURE 6:**
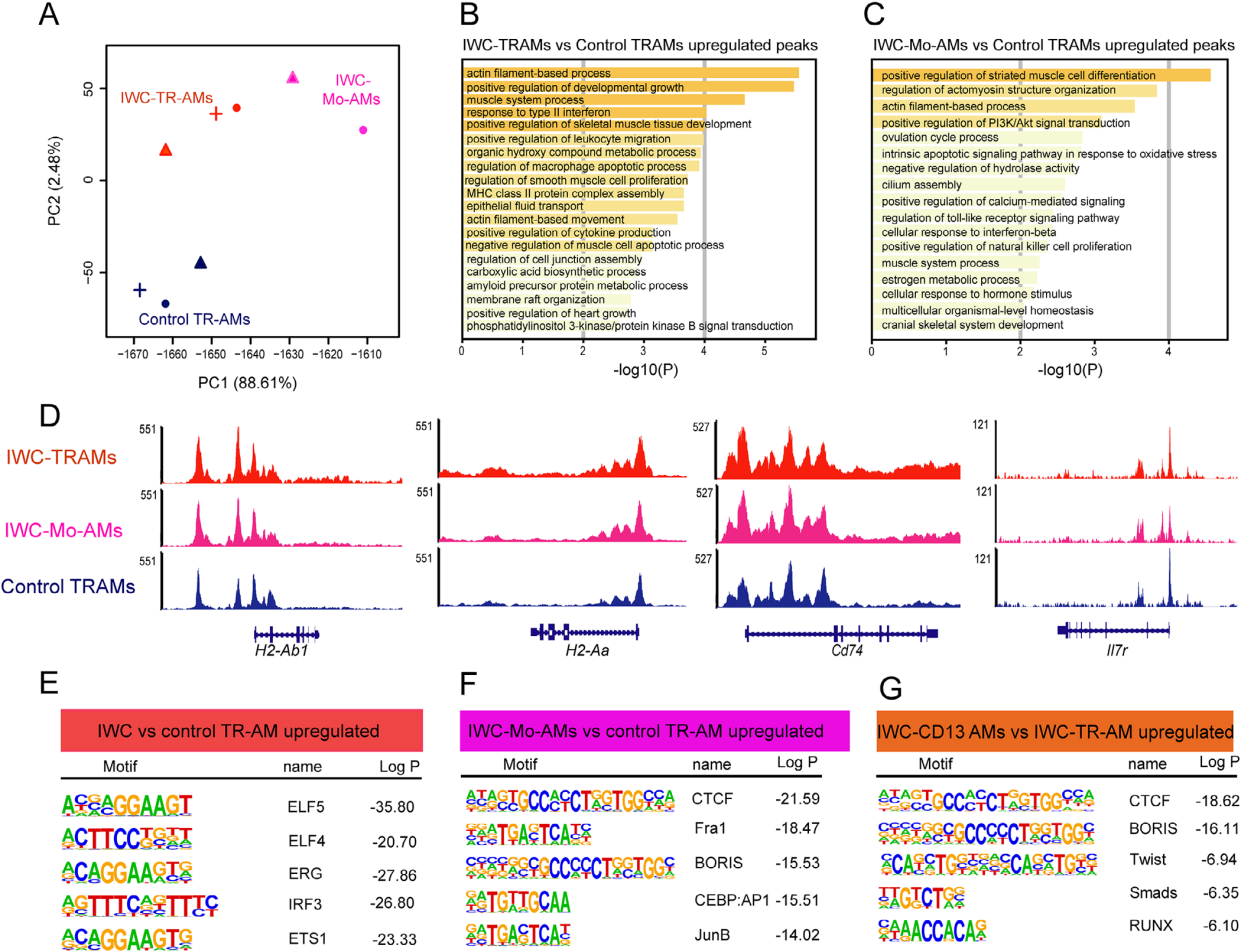
Epigenetic imprinting in IWC‐treated TR‐AMs and Mo‐AMs revealed by ATAC‐seq. (A) Principal component analysis (PCA) of ATAC‐seq data from IWC‐trained TR‐AMs, IWC‐trained Mo‐AMs, and control TR‐AMs. (B and C) Functional enrichment analysis of upregulated differentially accessible chromatin regions in (B) IWC‐trained TR‐AMs versus control TR‐AMs, and (C) IWC‐trained Mo‐AMs versus control TR‐AMs. Terms are ranked by −log_10_ (*p* value). (D) Integrative Genomics Viewer (IGV) tracks showing chromatin accessibility at representative immune‐related genes of *H2‐Ab1*, *H2‐Aa*, *Cd74*, and *IL7r*. (E–G) Transcription factor motif enrichment analysis of differentially accessible chromatin regions in (E) IWC‐trained TR‐AMs versus control TR‐AMs, (F) IWC‐trained Mo‐AMs versus control TR‐AMs, and (G) IWC‐trained Mo‐AMs versus IWC‐trained TR‐AMs. The top five enriched motifs are shown.

Trained immunity relies on stable epigenetic changes, so we used bulk ATAC‐seq to profile chromatin accessibility in TR‐AMs and Mo‐AMs derived from CD11B^+^CD13^+^ post‐IWC immunization. CD45.2^+^ mice were immunized intranasally with IWC, and TR‐AMs and CD11B^+^CD13^+^ Mo‐AMs were sorted from BALF at Day 5 post‐immunization. In parallel, control TR‐AMs were similarly sorted from unimmunized CD45.2^+^ mice. To ensure identical microenvironmental exposure prior to ATAC‐seq, all three populations were adoptively transferred into CD45.1^+^ recipients and re‐isolated at Day 25 post‐transfer by fluorescence‐activated cell sorting (FACS) (Figure S4A). Principal component analysis (PCA) showed distinct epigenetic profiles in IWC‐trained TR‐AMs and Mo‐AMs, clearly separated from control TR‐AMs (Figure 6A). Differential peak analysis identified 635 upregulated and 672 downregulated chromatin accessibility peaks in IWC‐trained TR‐AMs (Figure S4B), whereas IWC‐trained Mo‐AMs exhibited 1025 upregulated and 635 downregulated peaks (Figure S4C). Comparison between IWC‐trained Mo‐AMs and IWC‐trained TR‐AMs revealed 101 increased and 197 decreased peaks, suggesting different chromatin restructuring (Figure S4D). Functional enrichment analysis revealed that IWC‐trained TR‐AMs exhibited enrichment in pathways related to the Type II interferon response, positive regulation of leukocyte migration, MHC class II protein complex assembly, and positive regulation of cytokine production (Figure 6B). IWC‐trained Mo‐AMs were enriched in pathways related to PI3K/Akt signal transduction, Toll‐like receptor signaling, and enhancement of natural killer cell proliferation (Figure 6C). These results suggest that TR‐AMs and Mo‐AMs acquire distinct but complementary immune functions following IWC immunization, which is further supported by increased chromatin accessibility at key immune‐related genes, including *H2‐Ab1*, *H2‐Aa*, *Cd74*, *Il7r*, *S100a6*, *Tnfsf4*, *Il9*, and *Chil1* (Figure 6D and Figure S4E).

To identify TFs associated with these epigenetic changes, we performed motif enrichment analysis on the differentially accessible regions. In IWC‐trained TR‐AMs, we observed increased accessibility to binding motifs for the ETS family (ELF5, ELF4, ERG, ETS1), interferon regulatory factors (IRF3, IRF1, IRF2, IRF8, ISRE), and the bZIP family (NFIL3, CEBPA, CEBPB, Jun‐AP1) (Figure 6E). In IWC‐trained Mo‐AMs, motifs for the zinc finger family (CTCF, BORIS) and bZIP family (Fra1, CEBP:AP1, JunB) were preferentially enriched (Figure 6F). Comparative analysis between IWC‐trained Mo‐AMs and IWC‐trained TR‐AMs further revealed distinct motif enrichment, including C2H2 zinc finger TFs (CTCF, BORIS), bHLH TFs (Twist), MAD family TFs (Smad3), RUNX family TFs (RUNX), and nuclear receptor TFs (HNF4a) (Figure 6G). Together, these findings demonstrate that IWC immunization induces distinct epigenetic and TF motif signatures within the AM pool, which may be linked to enhanced and long‐lasting immune responsiveness of the trained AM pool.

### 2.7 Similar AM Pool Remodeling Is Observed With Other Bacterial IWC Vaccines

**FIGURE 7:**
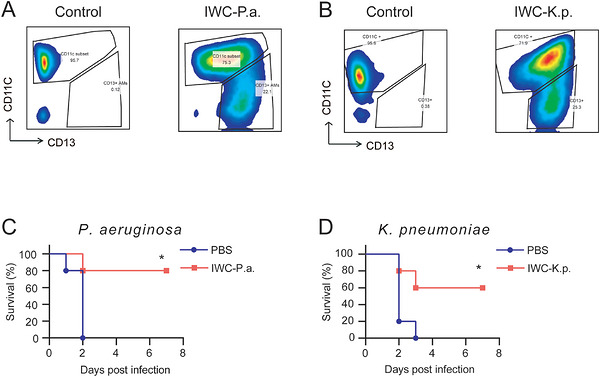
Vaccine‐induced CD11B^+^CD13^+^ Mo‐AMs in different MDR Gram‐negative bacteria infection models. (A and B) Representative flow cytometry dot plots showing CD11C and CD13 expression in BALF cells from mice immunized with (A) IWC from *P. aeruginosa* (IWC‐P.a.) or (B) IWC from *K. pneumoniae* (IWC‐K.p.) at Day 7 post‐immunization. (C and D) Kaplan–Meier survival curves of mice challenged with (C) *P. aeruginosa* or (D) *K. pneumoniae* at 30 days post‐immunization with the corresponding IWC vaccine (*n* = 5 per group). Survival was analyzed using the log‐rank test; ^*^ *p* < 0.05 versus control.

To explore whether similar AM pool remodeling is induced by IWC vaccines derived from other MDR Gram‐negative bacteria, we immunized mice with IWCs prepared from *P. aeruginosa* (IWC‐P.a.) or *Klebsiella pneumoniae* (IWC‐K.p.). Flow cytometry revealed that both vaccines elicited significant accumulation of CD11B^+^CD13^+^ Mo‐AMs and activation of TR‐AMs at 7 days post‐immunization (Figure 7A,B), showing a similar pattern of AM pool remodeling to that observed with MDR‐AB IWC.

We next assessed long‐lasting homologous protective efficacy in these vaccines. Mice were immunized intranasally with IWC‐P.a. or IWC‐K.p., then challenged intratracheally with a lethal dose of the homologous strain at 30 days post‐vaccination. Immunization resulted in 80% survival against *P. aeruginosa* (Figure 7C) and 60% survival against *K. pneumoniae* (Figure 7D). Together, these data indicate that similar AM pool remodeling occurs with multiple MDR Gram‐negative pathogen IWC vaccines.

## 3 Discussion

Conventional vaccine development has long focused on adaptive immunity, but the global crisis of MDR Gram‐negative bacteria—particularly MDR‐AB, a leading cause of lethal hospital‐acquired pneumonia—has highlighted the urgent need to harness innate immune memory. AMs, the frontline innate defenders of the pulmonary niche, exhibit heterogeneous responses to pathogens; yet how intranasal vaccination remodels the AM pool to establish long‐term immune protection remains poorly defined. Herein, our study fills this critical knowledge gap and demonstrates that intranasal IWC vaccination against *A. baumannii* orchestrates durable trained immunity via two synergistic and complementary cellular mechanisms: the transient recruitment and subsequent differentiation of Mo‐AMs, together with direct phenotypic, metabolic, and epigenetic reprogramming of TR‐AMs. This dual cellular programming endows the AM pool with sustained antimicrobial potency and immune hyperresponsiveness, thereby conferring long‐term protection against MDR‐AB infection. This working model expands the classical concept of trained immunity, which has historically been described as cell‐intrinsic functional priming within a single myeloid population, and provides a novel mechanistic paradigm for vaccine‐elicited trained innate immunity against drug‐resistant bacterial pulmonary infection.

Under physiological steady‐state conditions, the pulmonary AM pool is primarily sustained by long‐lived, self‐renewing TR‐AMs with a homeostatic and anti‐inflammatory phenotype. Our longitudinal transcriptomic and cytometric data collectively demonstrate that IWC vaccination triggers robust and rapid recruitment of a unique CD11B^+^CD13^+^ transitional Mo‐AM subset into the alveolar niche. This newly recruited population is distinguished by high expression of monocyte lineage and tissue remodeling‐associated genes, including *Itgam*, *Anpep* (CD13), *Cd14*, and *Il7r*. Notably, these vaccine‐recruited CD11B^+^CD13^+^ Mo‐AMs are not merely short‐lived inflammatory bystanders during acute vaccination responses. Multiple independent lineage‐tracing strategies, including bone marrow chimeras and congenic adoptive transfer models, rigorously validate that these Mo‐AMs persist in the alveolar microenvironment for at least 30 days post‐immunization and undergo progressive phenotypic maturation. During long‐term niche adaptation, CD11B^+^CD13^+^ Mo‐AMs gradually downregulate CD11B and CD13, and upregulate canonical TR‐AM signature molecules (CD11C), ultimately acquiring a TR‐AM‐like identity. These findings identify CD11B^+^CD13^+^ cells as a previously unrecognized transitional intermediate differentiation stage bridging circulating monocytes and mature AMs, which functionally replenishes and reshapes the long‐term AM pool following pulmonary vaccination (Figure 3). The stepwise maturation of recruited Mo‐AMs observed in our model partially recapitulates monocyte‐to‐AM differentiation during respiratory viral infection; however, our study further extends this concept by directly linking this transitional macrophage remodeling to vaccine‐induced trained immunity and long‐term antibacterial defense, highlighting a unique vaccination‐specific protective mechanism [3].

CD13 (encoded by *Anpep*, aminopeptidase N) is a conserved myeloid differentiation surface molecule that regulates macrophage and dendritic cell development, progenitor differentiation, and tissue immune homeostasis via both enzymatic activity and cell adhesion functions [23]. In this study, we identified stage‐specific dynamic CD13 expression as a reliable biomarker to trace monocyte recruitment and subsequent AM maturation following pulmonary IWC vaccination (Figure 2F). CD13 is highly enriched in vaccine‐elicited transitional Mo‐AMs, and its expression coincides with enhanced phagocytic capacity and pro‐inflammatory activation. Consistent with previous reports, CD13‐expressing myeloid cells exhibit superior microbial uptake, reactive oxygen species production, and tissue migration ability [24, 25, 26, 27], further supporting that CD13 marks a functionally hyperactive transitional macrophage subset critical for early bacterial clearance after vaccination.

Beyond the recruitment of Mo‐AMs, our scRNA‐seq analysis reveals that TR‐AMs undergo time‐dependent sequential phenotypic reprogramming after IWC stimulation, rather than remaining transcriptionally static. At the early stage (Day 7 post‐immunization), TR‐AMs are transcriptionally polarized toward an inflammatory and phagocytic‐activated state, characterized by the expansion of Cluster 3 enriched in pathogen clearance and pro‐inflammatory pathways. In the late phase (Day 60 post‐immunization), TR‐AMs further shift toward antigen presentation and sustained inflammatory regulatory profiles, dominated by antigen‐processing clusters (Cluster 2, high *H2‐Ab1*, *Cd74*) and inflammatory mediator‐secreting clusters (Cluster 4, high *Cxcl1*, *Tnf*). This temporal reprogramming enables the AM pool to coordinate immediate early antimicrobial defense and long‐term immune surveillance, which is particularly critical for restricting persistent and recurrent MDR Gram‐negative bacterial infection in immunocompromised and hospitalized populations.

Intriguingly, although the overall AM pool gradually returns to a quiescent CD11C^+^CD11B^−^ phenotypic landscape similar to naive TR‐AMs at 30 days post‐vaccination, this phenotypically convergent AM compartment is functionally and transcriptionally heterogeneous, consisting of unprimed resident TR‐AMs, in situ trained TR‐AMs, and long‐term differentiated Mo‐AMs (see Graphical Abstract Image). Such phenotypic convergence with stable functional immune memory represents a key cellular basis for the long durability of trained immunity, explaining why a single intranasal IWC vaccination is sufficient to establish protection lasting for at least 2 months.

Mechanistically, the establishment and maintenance of trained immunity rely on crosstalk between epigenetic chromatin remodeling and metabolic reprogramming. These two regulatory processes act together to sustain myeloid cells in a persistently hyperresponsive immune state. Our ATAC‐seq results confirm that IWC vaccination induces genome‐wide alterations in chromatin accessibility in both TR‐AMs and Mo‐AMs, stably imprinting a trained transcriptional landscape at immune‐related gene loci. These epigenetic alterations are closely coupled to metabolic rewiring that supports the heightened bioenergetic and biosynthetic demands of trained immunity and helps sustain long‐term innate immune memory. This integrated epigenetic‐metabolic axis determines the enhanced effector functions of vaccine‐trained AMs, including elevated TNF secretion, increased MHC II expression, and improved phagocytosis, all of which collectively promote MDR‐AB clearance (Figure 5) [28]. Enhanced MHC II expression on trained AMs also bridges innate and adaptive immune responses by promoting efficient T cell activation and priming [17, 29], while TNF reinforces antibacterial defenses consistent with previous infection models [30]. Furthermore, our adoptive transfer experiments provide direct functional evidence: isolated long‐term trained AMs from IWC‐immunized mice are sufficient to reduce pulmonary and systemic bacterial burdens in naive recipient mice upon MDR‐AB challenge, formally verifying that reprogrammed AMs serve as indispensable cellular mediators of vaccination‐conferred protection.

At the transcriptional regulatory level, comparative ATAC‐seq motif analysis uncovers subset‐specific epigenetic imprinting and TF dependency between TR‐AMs and Mo‐AMs, revealing divergent regulatory networks underlying their complementary functions. Both AM subsets display increased chromatin accessibility at antigen presentation and inflammatory cytokine gene clusters, consistent with the universal characteristics of trained innate immunity. Notably, the epigenetic signatures of IWC‐trained AMs show overlap with those reported in β‐glucan‐trained macrophages, suggesting potentially conserved regulatory features of innate immune memory across different stimuli [31, 32, 33]. TF motif analysis further highlighted subset‐specific programming: TR‐AMs exhibit preferential enrichment of binding motifs for ETS family TFs, IRF family members, and bZIP factors (CEBPβ, ATF1), which are well‐established regulators of macrophage activation and inflammatory response [34, 35, 36]. Several of these TFs, such as CEBPβ and ATF1, have been experimentally linked to trained immunity, supporting their potential role in vaccine‐induced TR‐AMs reprogramming [22, 33, 37, 38, 39]. In contrast, vaccine‐elicited Mo‐AMs display unique enrichment of C2H2 zinc finger factors (CTCF, BORIS) and distinct bZIP family members (Fra1, JunB), which modulate chromatin architecture and MHC II expression [40, 41]. Collectively, these data demonstrate that IWC vaccination triggers distinct yet coordinated epigenetic programs in TR‐AMs and Mo‐AMs, which correlate with their divergent phenotypic and functional roles.

Beyond *A. baumannii*, cross‐species validation shows that IWC vaccines derived from two clinically prevalent MDR Gram‐negative bacteria pathogens, *P. aeruginosa* and *K. pneumoniae*, trigger similar AM pool remodeling, accompanied by effective long‐term homologous protection. Given the lack of licensed anti‐MDR bacterial vaccines and limited treatment options for MDR bacterial pneumonia, these results highlight the potential value of AM‐directed trained immunity as a preventive strategy against MDR‐AB and other drug‐resistant respiratory pathogens. The ability of intranasal IWC vaccination to induce rapid, durable protection via innate immune modulation represents an attractive feature for at‐risk populations.

While our study characterizes vaccine‐induced remodeling of the AM pool, several limitations should be acknowledged and addressed in future investigations. First, the scRNA‐seq analysis was performed with moderate sequencing depth and pooled biological replicates, which may restrict the identification of rare cell subpopulations and subtle inter‐individual transcriptional heterogeneity. Although current datasets adequately resolve major AM subsets and core vaccine‐induced transcriptional alterations, higher sequencing depth, independent biological replicates, and larger cell capture numbers will help to dissect fine‐grained macrophage heterogeneity. Second, our study identifies correlative epigenetic signatures and key TFs motifs linked to IWC‐trained AM subsets, but the direct causal functions of candidate TFs (e.g., CTCF) in vaccine‐induced Mo‐AM differentiation and trained immunity remain unvalidated. To clarify their mechanistic roles, genetic approaches such as conditional gene knockout models or pharmacological interference strategies are required to experimentally confirm their functional relevance. Third, our fate‐mapping results support the differentiation of CD11B^+^CD13^+^ transitional Mo‐AMs into TR‐AM‐like cells; nevertheless, specific monocyte lineage genetic fate tracing models are needed to definitively clarify the long‐term origin and lifespan of these vaccine‐recruited macrophages. Fourth, all mechanistic and functional conclusions are established in murine experimental models; further translational verification using human primary AMs and clinical BAL samples from vaccinated individuals is essential to promote clinical transformation.

In conclusion, our study characterizes a dual‐origin remodeling mechanism of the AM pool triggered by intranasal IWC vaccination: the recruitment and progressive differentiation of transitional CD11B^+^CD13^+^ Mo‐AMs, combined with direct in situ epigenetic, metabolic, and functional reprogramming of resident TR‐AMs. This two‐tiered macrophage programming establishes long‐lasting defense against MDR‐AB pneumonia. Collectively, these findings advance our fundamental understanding of how respiratory vaccination reshapes pulmonary macrophage homeostasis and innate immune memory, and provide a mechanistic basis for the development of innate immunity‐targeted preventive strategies against MDR‐AB pneumonia.

## 4 Materials and Methods

### 4.1 Mice

C57BL/6 mice (6–8 weeks old) were purchased from GemPharmatech (Nanjing, China). *Rag1* gene knockout mice (Rag1^−/−^, B6.129S7‐Rag1^1Mom/J^) and age‐ and sex‐matched wild‐type control mice were purchased from the Model Animal Research Center of Nanjing University. Mice were housed under specific pathogen‐free conditions.

### 4.2 Bacterial Strains and Pneumonia Model

MDR‐AB strain LAC‐4 was kindly provided by Professor Xu and Professor Chen [42]. *A. baumannii* expressing GFP was constructed before [43]. MDR‐*P. aeruginosa* strain XN‐1 and MDR‐*K. pneumoniae* strain YBQ were isolated from Chongqing Southwest Hospital. The bacteria were grown in tryptone soy broth (for *A. baumannii*) or Luria Bertani broth (for *P. aeruginosa* and *K. pneumoniae*) at 37°C. At mid‐log‐phase, bacteria were collected and suspended in phosphate buffer saline (PBS). Fresh bacteria were used to infect the mice. For IWC preparation, fresh bacteria were fixed with 4% paraformaldehyde. Mice were immunized intranasally and challenged with bacteria as described before [20].

### 4.3 Induction of Trained Immunity in AMs With IWC In Vitro

Mice were euthanized, and the trachea was carefully exposed. An 18G catheter equipped with a 3‐way cock stop valve was inserted into the trachea. BALF was obtained by flushing the airways with 5 × 1 mL of sterile PBS containing 2 mM ethylenediaminetetraacetic acid (EDTA). BALF cells were cultured in DMEM (containing 10% fetal bovine serum [FBS] and 1% penicillin/streptomycin) in a culture plate for 1 h. Non‐adherent cells were discarded, and the remaining AMs were stimulated with *A. baumannii* IWC at a multiplicity of infection (MOI) of 1 for 24 h, followed by medium washout and further culture for 6 days.

### 4.4 scRNA‐seq

BALF was collected from control and IWC‐immunized mice at Day 7 and Day 60 post‐immunization. Cells were digested to a single‐cell suspension and viability was assessed using trypan blue exclusion, and equal numbers of viable cells from three independent biological replicates were pooled as one sample for each group. Then the cells were subjected to scRNA‐seq analysis using the BD Rhapsody system, following the manufacturer's instructions (BD Biosciences, New Jersey, USA). To minimize batch effects, cells from each group were labeled with unique sample tags using the mouse immune Single‐Cell Multiplexing Kit (BD Biosciences) and pooled to load onto a BD Rhapsody Cartridge for single‐cell mRNA capture using BD Rhapsody Cartridge Reagent Kit (BD Biosciences). Beads with captured mRNA were then retrieved from the cartridge into a single tube, and cDNA was synthesized on beads using BD Rhapsody cDNA Kit. The cDNA was split to construct the mRNA whole transcriptome analysis (WTA) library and the sample tag library using BD Rhapsody WTA Amplification Kit, following the BD Rhapsody protocol. The libraries were sequenced on Illumina nova6000 (Novogene Co. Ltd, Beijing, China) with a paired‐end 150 bp read length.

### 4.5 scRNA‐seq Data Analysis

The sequencing data were processed using BD Rhapsody analysis pipeline on Seven Bridges (SBG) (https://www.sevenbridges.com). Briefly, we utilized the BD Rhapsody WTA pipeline to analyze raw reads, aligning them to the GRCm38 (mm10) genome reference with sample multiplex selected of “Single‐Cell Multiplex Kit‐Mouse.” After demultiplexing and alignment, molecule count output files for each sample were imported into SeqGeq v1.8 software (BD biosciences) for downstream bioinformatics analysis. First, three samples were concatenated to a single dataset. Initial QC was performed to filter out low‐quality cells based on the following criteria: cells with fewer than 200 detected genes, greater than 20% mitochondrial gene expression, or a high number of unique molecular identifiers (UMIs) suggestive of doublets were excluded from further analysis. Dimensionality reduction was performed using the Seurat plugin in SeqGeq with default parameters, enabling the identification of clusters and DEGs. Unbiased UMAP was employed to visualize cluster distributions, with a resolution parameter of 0.5 used for initial clustering. DEGs were calculated in SeqGeq with *p* value < 0.05 and log2 fold‐change > 0.25 for upregulated genes, and heatmaps were generated within the software. Gene enrichment analysis was performed using the Metascape webtool (www.metascape.org) [44]. For subclustering, the AM cluster (G1) was gated and re‐clustered using the Seurat plugin in SeqGeq, following the same analytical framework as above.

### 4.6 Flow Cytometry Analysis

BALF cell suspensions were incubated with rat serum blocking at 4°C for 30 min to block Fc receptor. Fluorescence‐conjugated antibodies used in this study are provided in Table S3. Cell staining was performed with appropriate antibody cocktail in FACS buffer (2% FBS in PBS).

For intracellular TNF‐α staining, cells were first stained with surface markers in the dark at 4°C for 30 min. Cells were washed twice with washing buffer prior to fixation and permeabilization with the Foxp3/Transcription Factor Staining Buffer Set (Thermo Fisher Scientific, MA, USA) for 1 h at 4°C in the dark. Cells were incubated with rat serum blocking again, and then incubated with TNF‐α antibody. Cell death analysis was performed using Fixable Viability Stain 700 (FVS700) or DAPI, following the manufacturer's instructions (BD Biosciences). Appropriate Fluorescence Minus One (FMO) controls were included for gating strategy validation. Flow cytometry data were acquired on a BD LSRFortessa or BD FACSCanto II (BD Biosciences) and analyzed using FlowJo v10 (BD Biosciences). Cell populations were defined as follows: TR‐AMs: CD45^+^CD11B^−^CD11C^+^; monocytes: CD45^+^CD11B^+^Ly6C^+^; neutrophils: CD45^+^CD11B^+^Ly6G^hi^.

### 4.7 Fluorescence‐Activated Cell Sorting of AMs

BALF samples were harvested after IWC immunization. The cell suspension was filtered, centrifuged at 400 × g for 5 min at 4°C, and resuspended. Cells were counted and adjusted to 1 × 10^7^ cells/mL, followed by Fc receptor blocking. Next, 50 µL 2x staining mix containing anti‐CD45, anti‐CD11c, anti‐CD11B, anti‐CD13, anti‐Ly6C, and anti‐Ly6G was added to the samples, followed by incubation at 4°C for 30 min. The fluorescent labels of the antibodies were selected according to the sorting instrument for matching, and the antibodies are listed in Table S3.

Samples were washed with 1 mL FACS buffer and resuspended in 200 µL PBS containing DAPI Staining Solution (BD, #564907), incubated at room temperature for 5 min, and washed again. The cells were resuspended in 250 µL FACS buffer and sorted using BD FACSAria III or BD FACSAria Fusion (BD Biosciences). The instrument was calibrated, and doublets were excluded using forward and side scatter gating. Cells were gated by FSC/SSC, and dead cells were excluded by DAPI staining. The CD11B^−^CD11C^+^ and CD11B^+^CD13^+^ cell populations were sorted into tubes with ice‐cold DMEM with 5% FBS. Following sorting, a small aliquot was reanalyzed to confirm purity (≥ 90%), and viability was assessed using trypan blue staining (> 80%).

### 4.8 Real‐Time PCR

Total RNA of cells was extracted using RNA iso Plus (omega BIO‐TEK, R6831‐02, USA) and reverse transcribed to cDNA with PrimeScript RT reagent Kit (Takara Biotechnology). Gene expression was detected using SYBR Green Premix (Takara Biotechnology) on a CFX96qRT‐PCR detection machine (Bio‐Rad, Hercules, CA, USA) with specific primers listed in Table S4. The ΔΔCt method was used to calculate the relative gene expression with β‐actin as the housekeeping gene.

### 4.9 Cytospin and Giemsa Staining

Various AM subsets were FACS‐sorted into PBS. Afterward, cells were centrifuged onto glass slides using cytocentrifugation (Thermo Fisher Scientific, Waltham, MA). Slides were stained following the May‐Grünwald/Giemsa method, and images were acquired using an optical microscope (CX‐21; Olympus, Tokyo, Japan). Images are shown with a 400x magnification.

### 4.10 Simultaneous Intranasal and Intravascular Antibody Labeling

Mice were anesthetized with isoflurane and injected intratracheally with 1 µg BV605 conjugated anti‐CD45 antibody (clone number 30‐F11, BD Biosciences) in 20 µL PBS. Then, mice were injected intravenously with 1 µg PE‐Cy7 conjugated anti‐CD45 antibody (clone number 104, BD Biosciences) in 100 µL PBS. After a 30‐min interval, the mice were euthanized, and lung cells were collected. The lungs were minced to generate single‐cell suspensions, and flow cytometry was used to measure the percentages of PE‐Cy7^+^ or BV605^+^ cells within different cell populations.

### 4.11 Labeling of TR‐AMs With PKH26‐PCL

PKH26‐phagocytic cell labeling kit (PCL; Sigma‐Aldrich, St. Louis, MO) was used to label TR‐AMs selectively. Briefly, PKH26‐Phagocytic Cell Linker was diluted in Diluent B (30 µM) as per the manufacturer's instructions and instilled intratracheally into the airways of anesthetized mice 5 days before IWC immunization. Subsequently, BALF cells were harvested at various time points post immunization and subjected to flow cytometry. PKH26^+^ cells were identified as TR‐AMs, and PKH26^−^ cells were regarded as recruited cells.

### 4.12 Bone Marrow Chimeras With Lung Shielding

Bone marrow chimeras with lung shielding were generated to assess the origin and fate of CD11B^+^CD13^+^ cells. C57BL/6 recipient mice (CD45.2^+^) were anesthetized and placed between lead strips (3‐cm thick by 10‐cm wide) to protect the lungs from lethal irradiation (8 Gy γ‐radiation). Bone marrow cells (6 × 10^6^) were isolated from CD45.1^+^ donor mice and intravenously injected into recipient CD45.2^+^ C57BL/6 within 2 h post‐irradiation. For the subsequent 2 weeks, the mice were provided with autoclaved water containing 50 µg/mL of gentamicin, followed by a switch to standard housing conditions. Six weeks post‐transplantation, bone marrow chimera mice were immunized intranasally with IWC. TR‐AMs (CD45.2^+^ cells) and Mo‐AMs (CD45.1^+^ cells) in BALF at Day 7 or Day 30 post‐immunization were analyzed by flow cytometry.

### 4.13 Adoptive Transfer of AMs

To test the fate of AMs, TR‐AMs and CD11B^+^CD13^+^ cells were FACS‐sorted from C57BL/6 WT (CD45.2^+^) donor mice at Day 5 following IWC immunization. These cells were adoptively transferred intratracheally into congenic CD45.1^+^ recipient mice. On Day 25 post‐transfer, engrafted cells were analyzed for the surface marker expression of CD11C, CD11B, and CD13 using flow cytometry. To test the function of IWC‐immunized AMs, CD11C^+^CD11B^−^ AMs were FACS‐sorted from BALF of IWC‐immunized or control mice at Day 30 post‐immunization (purity > 95%). A total of 5 × 10^4^ donor AMs were intratracheally transferred into the airway of anesthetized recipient mice. Four hours post‐transfer, recipient mice were challenged with MDR‐AB LAC‐4 (5 × 10^6^ CFU). At 24 h post‐challenge, mice were sacrificed, and lungs were homogenized in PBS. Bacterial burdens were quantified by plating serial dilutions of lung homogenates on tryptone soy agar.

### 4.14 Phagocytosis Assay

For in vivo evaluation of phagocytosis, mice were immunized intranasally with IWC for 7 or 30 days, then infected intratracheally with GFP‐expressing *A. baumannii* (1 × 10^7^). After 2 h, BALF cells were collected, and the percentages of phagocytic macrophages (percentage of GFP^+^ cells among the AMs) in different cells were measured by flow cytometry. For in vitro phagocytosis assay, AMs of control mice were stimulated with IWC (MOI of 1） for 24 h. After removal of stimulants, cells were continuously cultured for 7 days, followed by infection with GFP‐*A. baumannii* (MOI of 10) for 2 h at 37°C. The frequency of phagocytic AMs was determined by flow cytometry 2 h after the in vitro infection.

### 4.15 Glycolytic Activity Analysis

Glycolytic function in AMs was quantified using an Agilent Seahorse XFe96 Analyzer (Agilent Technologies, Santa Clara, CA, USA) with the Seahorse XF Glycolytic Rate Assay Kit (Agilent), following the manufacturer's optimized protocol and established methodologies. This assay measures glycolytic proton efflux rate (glycoPER, pmol H^+^/min), a precise metric of glycolysis that corrects total extracellular acidification for mitochondrial CO_2_‐derived protons. AMs were isolated from C57BL/6 mice across three experimental groups: Control, IWC‐7d (CD11B^−^CD11C^+^ TR‐AMs and CD11B^+^CD13^+^ Mo‐AMs), and IWC‐30d (CD11B^−^CD11c^+^ TR‐AMs). Cells were FACS‐sorted as described above. Twenty‐four hours before the assay, 200 µL/well of XF Calibrant was added to the XFe96 sensor cartridge and incubated overnight at 37°C in a non‐CO_2_ incubator. XF base medium (Agilent) was supplemented with 1 mM sodium pyruvate, 2 mM L‐glutamine, and 10 mM D‐glucose, pH‐adjusted to 7.4 ± 0.05, and pre‐warmed to 37°C. Sorted viable AMs were washed twice in pre‐warmed assay medium, resuspended, and seeded at 8 × 10^4^ cells/80 µL/well in a 96‐well XF cell culture microplate, with five technical replicates per group. Plates were centrifuged at 200 × g for 5 min to facilitate cell adherence, then 100 µL/well of assay medium was added. Cells were incubated for 45 min at 37°C in a non‐CO_2_ incubator before the assay. During the assay, rotenone/antimycin A (Rot/AA, final concentration 0.5 µM, to inhibit mitochondrial respiration) was loaded into Port A, and 2‐deoxy‐D‐glucose (2‐DG, final concentration 50 mM, to block glycolysis) was loaded into Port B of the sensor cartridge. The Glycolytic Rate Assay program was run via Wave software (v2.6, Agilent), with real‐time measurement of ECAR. glycoPER was automatically calculated by the Wave software to derive basal glycoPER, compensatory glycoPER, and non‐glycolytic acidification. All data were normalized to cell number and analyzed using Wave.

### 4.16 ATAC‐seq

C57BL/6 CD45.2^+^ mice were immunized intranasally with *A. baumannii* IWC. At Day 5 post‐immunization, CD45.2^+^ TR‐AMs and CD11B^+^CD13^+^ cells were FACS‐sorted from BALF and separately transferred intratracheally into congenic CD45.1^+^ recipient mice. At Day 25 post‐transfer, BALF was collected, and engrafted CD45.2^+^ cells were re‐isolated by FACS sorting. The sorted population included: CD45.2^+^ IWC‐immunized TR‐AMs (3 biological replicates), CD45.2^+^ IWC‐immunized CD11B^+^CD13^+^ cells‐derived Mo‐AMs (2 biological replicates), and CD45.2^+^ TR‐AMs from control mice (3 biological replicates). Cells were washed with cold PBS, and viability (> 85%) was assessed via a trypan blue exclusion assay before ATAC‐seq library preparation.

ATAC‐seq was performed by Shanghai Jiayin Biotechnology Co. Ltd. Briefly, nuclei were obtained from single‐cell suspensions following an established ATAC‐seq protocol [45]. A total of 40,000 nuclei per sample were subjected to transposition using Tn5 transposase (Illumina). Transposed DNA fragments were purified using the MinElute PCR Purification Kit (Qiagen). PCR amplification of transposed DNA was performed using 1X NEBNext High‐Fidelity PCR Master Mix (New England Biolabs, MA). Amplified libraries were purified using the MinElute PCR Purification Kit (Qiagen). Libraries were prepared using barcoded primers and further purified before sequencing on the Illumina NovaSeq 6000 platform with paired‐end 150 bp reads.

### 4.17 Bioinformatics Analysis of ATAC‐seq Data

Following sequencing, raw sequencing reads were filtered using Trimmomatic to remove adapter sequences and low‐quality reads, followed by quality assessment via FastQC. Filtered reads were aligned to the mouse reference genome (mm10) using Burrows‐Wheeler Aligner (BWA), generating Binary Alignment/Map (BAM) files from uniquely mapped reads. ATAC‐seq peak regions were identified using MACS2 with a q‐value cutoff < 0.05. Peaks were annotated using the annotatePeak function in ChIPseeker, and target genes for peaks were assigned based on peak proximity to the transcription start site (TSS) (distance to TSS < 100,000 bp). To assess sample correlations and identify potential outliers, PCA was performed on merged peak signals from all samples using plotPCA, ranking principal components by variance. Differentially accessible peaks were identified using DESeq2 (threshold: |log_2_FC| > 1, *p* < 0.05). TF binding motifs were analyzed using HOMER's findMotifsGenome.pl, using peak sequences extracted from the genome FASTA file. Peak accessibility for key genes was visualized via Integrative Genomics Viewer (IGV) [46]. Functional enrichment of differentially accessible peaks was performed using Metascape (www.metascape.org).

### 4.18 Stimulation of AMs Ex Vivo

Mice were immunized intranasally with IWC or PBS as control. AMs (CD11c^+^) were sorted by FACS from BALF at Day 30 post‐immunization and stimulated ex vivo with IWC (MOI = 1) for 2 h, and supernatants were collected for measuring TNF‐α using ELISA.

### 4.19 ELISA

TNF‐α, IL‐6, and IL‐1β concentrations in serum, lung homogenates, and cell culture supernatants were detected using a mouse TNF‐α ELISA kit, a mouse IL‐6 ELISA kit, and a mouse IL‐1β ELISA kit (eBioscience, San Diego, CA, USA) following the manufacturer's instructions.

### 4.20 Statistical Analysis

Statistical analyses were performed using GraphPad Prism 8. Bacterial burdens (log_10_ CFU) were expressed as geometric mean ± geometric standard deviation (SD) with individual biological replicates, and comparisons were conducted via the Mann–Whitney *U* test. Other bar graph data were presented as means ± SD. Survival data were compared by log‐rank test. Two normally distributed sample groups were compared using Student's *t*‐test. For grouped data, statistical significance was evaluated by ordinary two‐way ANOVA. All comparisons used a two‐sided *α* of 0.05 for significance testing, and *p* < 0.05 was considered significant. Detailed statistical approaches for each dataset are specified in the corresponding figure legends.

## Author Contributions

Y.S. handled conceptualization, funding acquisition, and manuscript review and editing. X.M.Z., Y.Y.Z., C.Y.X., and N.W. performed the major experiments and collected and analyzed experimental data. Y.L., K.C., Y.X., H.Y., and X.C.S. assisted with sample preparation, data validation, and manuscript revision. All authors read and approved the final manuscript.

## Funding

This research was funded by the National Natural Science Foundation of China (NSFC) (Grant No. 32270944), Prevention and Control of Emerging and Major Infectious Diseases—National Science and Technology Major Project of China (Grant No. 2025ZD01903305), and the 1·3·5 Project for Disciplines of Excellence, West China Hospital, Sichuan University (No. ZYXY21004).

## Ethics Statement

The animal protocols adhered to the National Institutes of Health Guide for the Care and Use of Laboratory Animals and were approved by the Institutional Animal Care and Use Committee of West China Hospital, Sichuan University (Approval No. 2019190A).

## Conflicts of Interest

The authors declare no conflicts of interest.

## Supporting information



Document S1. **Figure S1–S4** and **Table S3‐S4**
**Table S1**: Excel file containing data showing the signature genes of each cluster of TR‐AMs.
**Table S2**: Excel file containing data showing the signature genes of each cluster of IWC‐treated TR‐AMs in vitro.

## Data Availability

Single‐cell RNA‐seq data (GEO: GSE167359) and ATAC‐seq data (GEO: GSE287269) have been deposited at Gene Expression Omnibus (GEO) and are publicly available as of the date of publication. This study does not report original code. Further information and requests for resources and reagents should be directed to the lead contact, Yun Shi (shiyun@wchscu.cn).
