# Supplementary material for: Vaccination Drives Alveolar Macrophages Pool Remodeling via Training Resident Cells and Recruiting Monocyte‐Derived Cells to Defend Against Multidrug‐Resistant Acinetobacter baumannii
Source: MedComm (2020). 2026 Aug 3;7(8):e70887. doi: 10.1002/mco2.70887 (PMC13430501; doi:10.1002/mco2.70887)
Supplement: Supplementary file 1 — Document S1. Figure S1–S4 and Table S3‐S4 Table S1: Excel file containing data showing the signature genes of each cluster of TR‐AMs. Table S2: Excel file containing data showing the signature genes of each cluster of IWC‐treated TR‐AMs in vitro. [file MCO2-7-e70887-s001.docx]

Supplemental materials

**Vaccination Drives Alveolar Macrophage Pool Remodeling via Training Resident Cells and Recruiting Monocyte-Derived Cells to Defend Against Multidrug-Resistant *Acinetobacter baumannii***

Running title: Vaccination remodels Alveolar Macrophage Pool.

Xiaomin Zhang,† Yangyang Zhou, † Chuanying Xiang,† Ning Wang,† Yan Li, Kai Chen, Yu Xie, Hong Yang, Xiangcheng Sun, Yun Shi*

**Affiliations:**

Institute of Biopharmaceuticals, West China Hospital, Sichuan University, Chengdu, Sichuan 610041, China;

† these authors contribute equally.

*Correspondence: [shiyun@wchscu.cn](mailto:shiyun@wchscu.cn) (YS); Tel: +086-18375739219

**Figure S1**

**c**

Figure S2


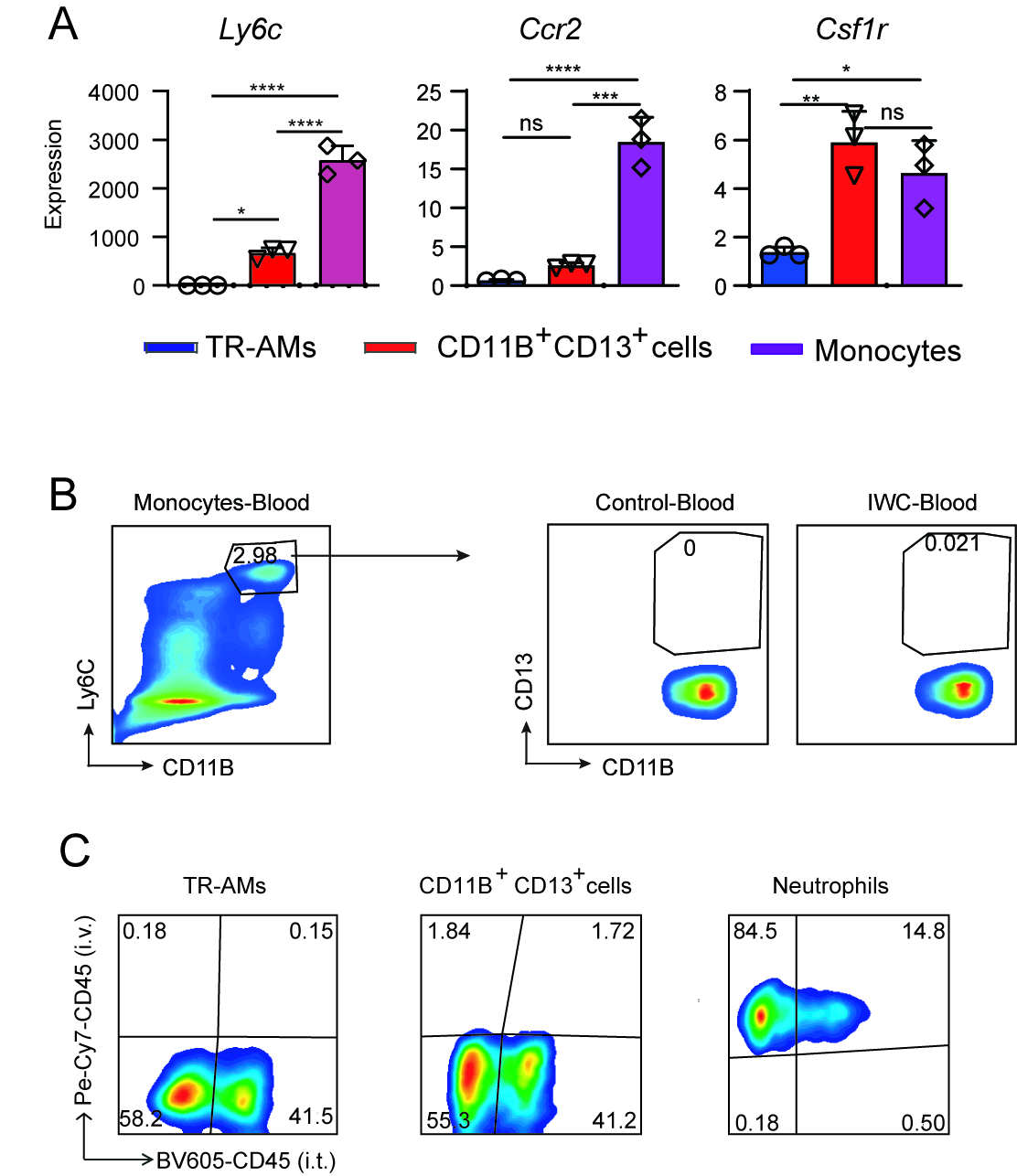


**Figure S2. Molecular and spatial characterization of CD11B⁺CD13⁺ Mo-AMs. Related to Figure 3.**

(A) Real-time PCR analysis of monocyte lineage and macrophage differentiation markers (Ly6c2, Ccr2, and Csf1r) in FACS-sorted CD11B⁻CD11C⁺ TR-AMs, CD11B⁺CD13⁺ cells from BALF, and circulating monocytes (developmental reference control) at day 7 post-intranasal IWC immunization. Data are expressed as mean ± SD. *, P<0.05; **, P<0.01; ***, P<0.001; ****, P<0.0001 (one-way ANOVA). ns, not significant.

(B) Flow cytometric analysis showing CD13 expression on circulating monocytes (CD11B⁺Ly6C⁺) from control and IWC-immunized mice show minimal CD13 expression in both groups.

(C) In vivo CD45 labeling assay to assess the alveolar localization of CD11B⁺CD13⁺ cells. Mice received intravenous PE-Cy7-conjugated anti-CD45 to label circulating cells and intratracheal BV605-conjugated anti-CD45 to label airway-resident cells. Representative flow cytometry plots show that both TR-AMs and CD11B⁺CD13⁺ cells are predominantly BV605⁺, confirming their localization within the alveolar space. Neutrophils served as a control for circulating CD45⁺ cells.

**Figure S3**

**
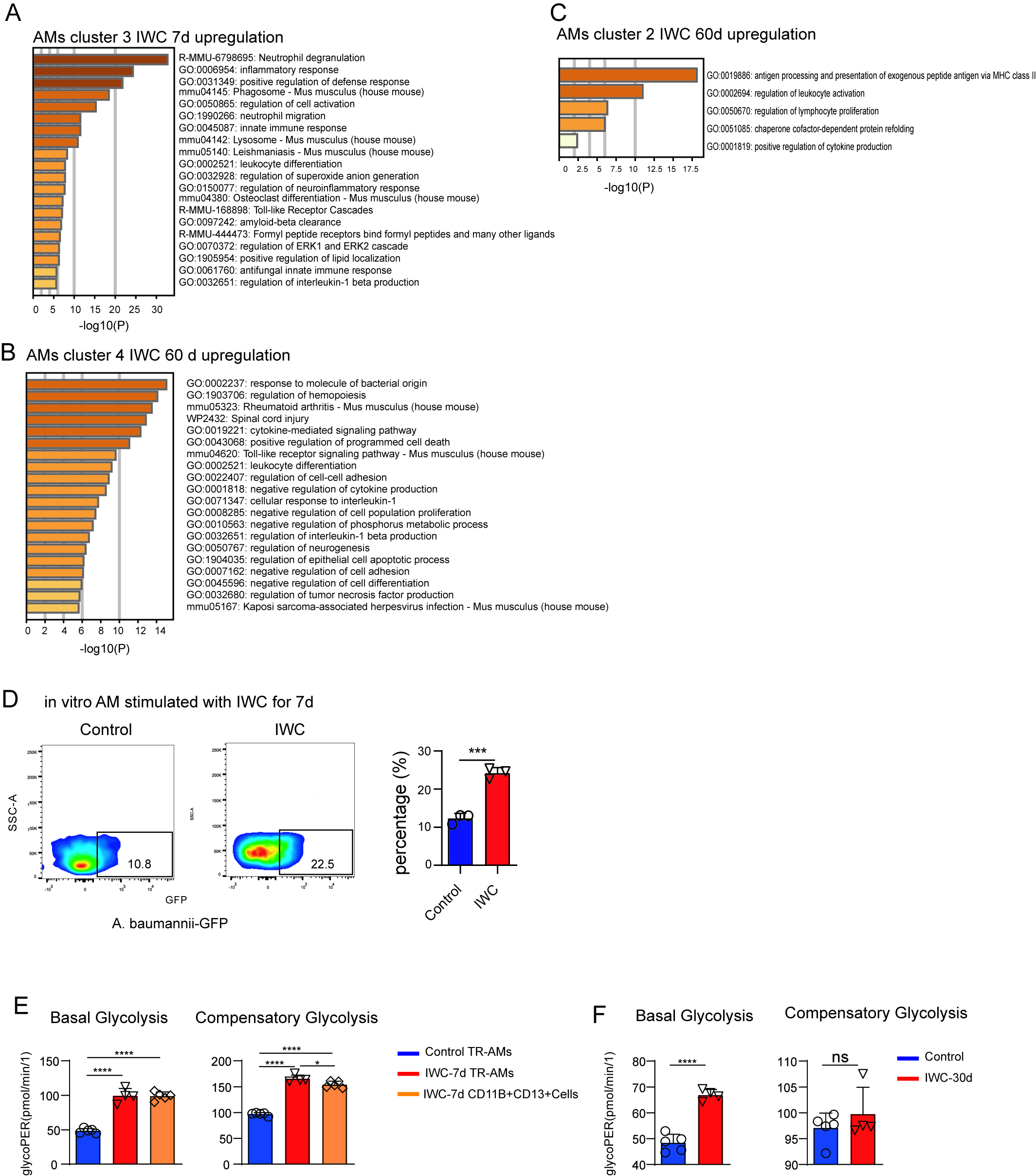
Figure S3. Functional Reprogramming of AMs Following IWC Immunization.** Related to Figure 5.

(A-C) Gene set enrichment analysis of the signature genes of cluster 3 at day 7 (A) and cluster 2 (B) and cluster 4 (C) at day 60 of IWC-treated TR-AMs.

(D) Flow cytometry detecting the phagocytic ability of IWC-trained TR-AMs in vitro.

(E) Basal and compensatory glycolysis of Control TR-AMs, IWC-trained TR-AMs, and IWC-induced CD11B⁺CD13⁺ Mo-AMs were analyzed for glycoPER (pmol/min/1) at day 7 post-IWC immunization.

(F) Basal and compensatory glycolysis of control TR-AMs and IWC-trained TR-AMs at day 30 post-immunization. Glycolytic activity was measured by glycoPER (pmol/min/1). Statistically significant differences are indicated as *****P* < 0.0001 and **P* < 0.05; ns, not significant.

**Figure S4**

###
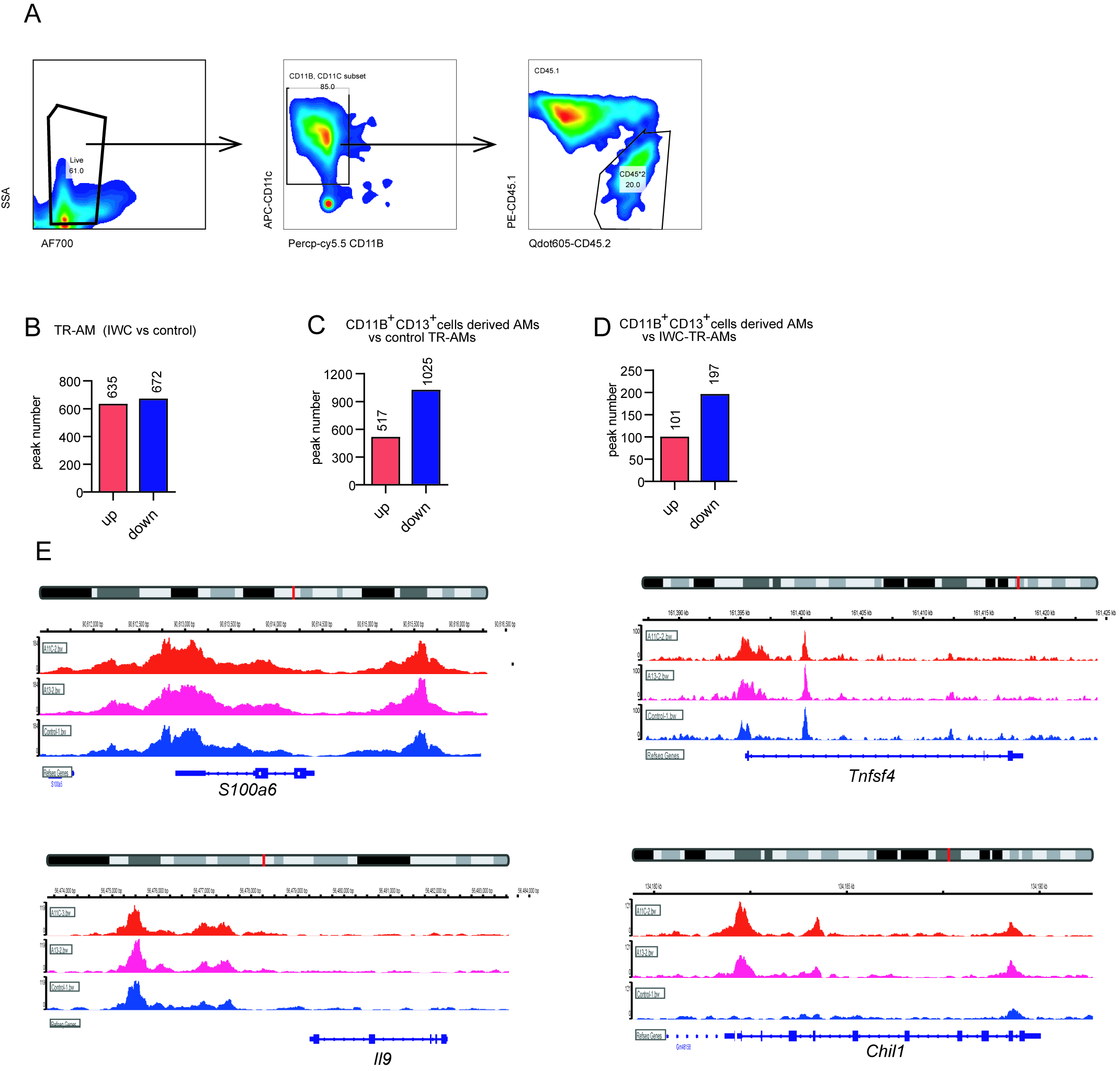
Figure S4. Chromatin Accessibility Reprogramming in IWC-Trained AMs pool. Related to Figure 6.

### Flow cytometry showing the gating strategy for sorting CD45.2+ cells back 25d post-transfer for ATAC-seq.

### (B-D) Differential peak numbers in IWC-trained TR-AMs compared to control TR-AMs (B), CD11B^+^CD13^+^ cells-derived Mo-AMs compared to control TR-AMs (C), and CD11B^+^CD13^+^ cells-derived Mo-AMs to IWC immunized TR-AMs (D).

### (E) Integrative Genomics Viewer tracks of ATAC-seq data showing the chromatin accessibility for representative genes of S100a6, Tnfsf4,Il9, and Chil1.

Table S3. Antibodies used in this study.

| Antibody | Fluorophore-conjugation | Clone | Distributor |
| --- | --- | --- | --- |
| Anti-mouse CD45.2 | BV605 | 104 | BioLegend |
| Anti-mouse CD45.1 | PE | A20 | BioLegend |
| Anti-mouse CD13 | BV421 | R3-242 | BD Bioscience |
| Anti-mouse CD13 | FITC | R3-242 | BD Bioscience |
| Anti-mous MHCII | BV510 | M5/114.15.2 | BD Bioscience |
| Anti-mouse CD11C | APC | N418 | BioLegend |
| Anti-mouse CD11C | BV510 | N418 | BioLegend |
| Anti-mouse CD11C | PE | N418 | BioLegend |
| Anti-mouse LY6C | Alexa Fluor® 647 | HK1.4 | BioLegend |
| Anti-mouse LY6C | PE-Cy7 | HK1.4 | BioLegend |
| Anti-mouse LY6C | Alexa Fluor® 700 | HK1.4 | BioLegend |
| Anti-mouse LY6G | APC-Cy7 | 1A8 | BioLegend |
| Anti-Human/mouse CD11B | PerCP-Cy5.5 | M170 | BioLegend |
| Anti-mouse TNFα | PE-Cy7 | MP6-XT22 | eBioscience |
| Anti-mouse CD45 | BV605 | 30-F11 | BD Biosciences |
| anti-mouse CD45 | PE-cy7 | 104 | BD Biosciences |
| Anti-mouse CD45 | PE | 30-F11 | BioLegend |
| Anti-mouse CD127 | BV711 | SB/199 | BD Bioscience |
| Anti-mouse Siglec-F | BV421 | E50-2440 | BD Bioscience |
| DAPI | - | #564907 | BD Bioscience |
| Fixable Viability Stain 700 | - | #564997 | BD Bioscience |

Table S4. Primers used in this study.

| Gene name | Forward primer | Reverse primer |
| --- | --- | --- |
| Itgam | CCATGACCTTCCAAGAGAATGC | ACCGGCTTGTGCTGTAGTC |
| Anpep | ACACCACCTCCACCATCATCAG | TCCTCCACCGTAATTCTCAAACAG |
| Apoe | TGGAAAGCAGGACTTAGCCG | CCATCAGTGCCGTCAGTTCT |
| Il7r | GCGGACGATCACTCCTTCTG | AGCCCCACATATTTGAAATTCCA |
| Cxcr4 | GACTGGCATAGTCGGCAATG | AGAAGGGGAGTGTGATGACAAA |
| Itgax | CTGGATAGCCTTTCTTCTGCTG | GCACACTGTGTCCGAACTCA |
| Chil3 | CAGGTCTGGCAATTCTTCTGAA | GTCTTGCTCATGTGTGTAAGTGA |
| Siglecf | CCCTGCCTCCACCATAAATGA | CATAGTGGAGTTCAGGCTCATC |
| Mrc1 | CCCAAGGGCTCTTCTAAAGCA | CGCCGGCACCTATCACA |
| Marco | GAAGACTTCTTGGGCAGCAC | GTGAGCAGGATCAGGTGGAT |
| Tnf | CCTATGTCTCAGCCTCTTCTCAT | CACTTGGTGGTTTGCTACGA |
| Ccr2 | GCTGTGTTTGCCTCTCTACCAG | CAAGTAGAGGCAGGATCAGGCT |
| Csf1r | TGGATGCCTGTGAATGGCTCTG | GTGGGTGTCATTCCAAACCTGC |
| Ly6C | GCGCCTCTGATGGATTCTGCAT | ATCCCTGATTGGCACACCAGCA |
| Il6 | CCTCTCTGCAAGAGACTTCC | CTCCGGACTTGTGAAGTAGG |
| Il1β | GGACCCCAAAAGATGAAGGGCTGC | GCTCTTGTTGATGTGCTGCTGCG |
| β-actin | GGCTGTATTCCCCTCCATCG | CCAGTTGGTAACAATGCCATGT |
| Cd74 | ATGACCCAGGACCATGTGATG | CCCTTCAGCTGCGGGTACT |
| H2-Ab1 | CATGGGCGAGTGCTACTTCA | CGACATTGGGCTGTTCAAGC |
| Cxcl1 | ATGGCTGGGATTCACCTCAA | AGTGTGGCTATGACTTCGGT |
| S100a6 | CCTTCTCGTGGCCATCTT | CCCAGGAAGGCGACATAC |
